# Collateral thyroid damage: A case report of suspected TKI-induced hypothyroidism

**DOI:** 10.1177/2050313X261431592

**Published:** 2026-03-19

**Authors:** Adam Stenman, Renske Altena, Henrik Falhammar, Eva Darai-Ramqvist, Carl Christofer Juhlin

**Affiliations:** 1 Department of Breast, Endocrine Tumors and Sarcoma, Karolinska University Hospital, Stockholm, Sweden; 2 Department of Molecular Medicine and Surgery, Karolinska Institutet, Stockholm, Sweden; 3 Department of Oncology-Pathology, Karolinska Institutet, Stockholm, Sweden; 4 Department of Endocrinology, Karolinska University Hospital, Stockholm, Sweden; 5 Department of Pathology and Cancer Diagnostics, Karolinska University Hospital, Stockholm, Sweden

**Keywords:** TKI, adverse drug reaction, thyroiditis, case report

## Abstract

Tyrosine kinase inhibitors (TKIs) are widely used in cancer therapy and are known to induce hypothyroidism in some patients, though the mechanisms remain unclear. We describe a 58-year-old man with metastatic gastrointestinal stromal tumor who developed clinical hypothyroidism during TKI treatment. Imaging revealed thyroid enlargement, and fine-needle aspiration yielded indeterminate cytology. The patient subsequently underwent thyroid lobectomy, which revealed a unique pattern of thyroiditis with multiple hyaline nodules containing entrapped follicular cells and scattered immune cell infiltration. Postoperatively, the patient was in a good clinical condition, but persistently elevated thyroid-stimulating hormone concentrations despite normal T4 concentrations were likely due to pre-existing TKI-induced thyroid dysfunction limiting compensatory function after hemithyroidectomy. The above-mentioned histological presentation is atypical for conventional thyroiditis and may represent a distinct pathological response associated with TKI therapy. The clinical, cytological, and histological findings in this case suggest a potential link between TKIs and thyroid dysfunction, highlighting the need for increased awareness of thyroid complications during targeted therapy. This case underscores the importance of considering iatrogenic causes in new-onset hypothyroidism in oncology patients and suggests that distinct histological features may serve as clues to the underlying pathogenesis.

## Introduction

Tyrosine kinase inhibitors (TKIs) are commonly used to treat various types of malignancies, and a common side effect is the induction of hypothyroidism through mechanisms that are only partly understood and poorly characterized in humans. By competing with adenosine triphosphate for binding sites on various tyrosine kinases, TKIs effectively curb abnormal activation and the subsequent downstream intracellular signaling pathways linked to cellular proliferation. The family of receptors employing tyrosine multikinase signaling encompasses a diverse array of proteins influencing tumor growth, among them platelet-derived growth factor receptor, epidermal growth factor receptor, and vascular endothelial growth factor receptor (VEGFR), among others. A large meta-analysis of previously published cases revealed that one-third of patients treated with TKIs experienced thyroid dysfunction as a result of their treatment, particularly hypothyroidism.^1^ Currently approved TKIs have different prevalence of thyroid toxicity, with sunitinib and sorafenib emerging as the most frequent cause of hypothyroidism. The exact mechanism driving TKI-induced hypothyroidism is not well understood, with one explanation relating to decreased vascular density in the thyroid gland as a result of the anti-angiogenic effect of TKIs. The thyroid gland is highly vascularized, and follicular epithelial cells express VEGFR.^2^ Notably, administration of VEGFR inhibitors to mice led to a significant reduction in thyroidal blood vessels.^3^ Among other proposed pathogenic theories, the anti-peroxidase activity of TKIs (similar to that of anti-thyroid drugs such as propylthiouracil) is acknowledged.^4^ Additional mechanisms have been proposed beyond reduced thyroid vascularity and anti-peroxidase effects. A recurring biochemical pattern during TKI therapy is an increase in serum thyroid-stimulating hormone (TSH) concentrations accompanied by a disproportionate reduction in circulating triiodothyronine (T3) relative to thyroxine (T4) concentrations, resulting in a decreased T3:T4 ratio. This phenotype suggests that TKIs can interfere not only with the thyroid gland itself, but also with peripheral thyroid hormone homeostasis.^5^ In particular, accumulating evidence indicates that TKIs affect deiodinase-mediated thyroid hormone metabolism in healthy tissues, with reduced type 2 deiodinase activity and increased type 3 deiodinase activity contributing to reduced generation and increased inactivation of T3, respectively, and to higher reverse T3 concentrations. TKIs have also been reported to impair cellular thyroid hormone uptake by non-competitively inhibiting thyroid hormone transporters, which could further contribute to elevated TSH via altered hypothalamic–pituitary feedback. Collectively, these effects provide a plausible explanation for fluctuating thyroid function tests during TKI treatment and emphasize that TKI-associated hypothyroidism may reflect combined thyroidal and extrathyroidal mechanisms rather than isolated primary thyroid failure.

However, even though several pathogenic mechanisms potentially underlying the disease have been explored, histological descriptions detailing the microscopic appearance of thyroid glands from patients with thyroid dysfunction in relation to TKI treatment are largely lacking.

## Case presentation

**Figure 1.**
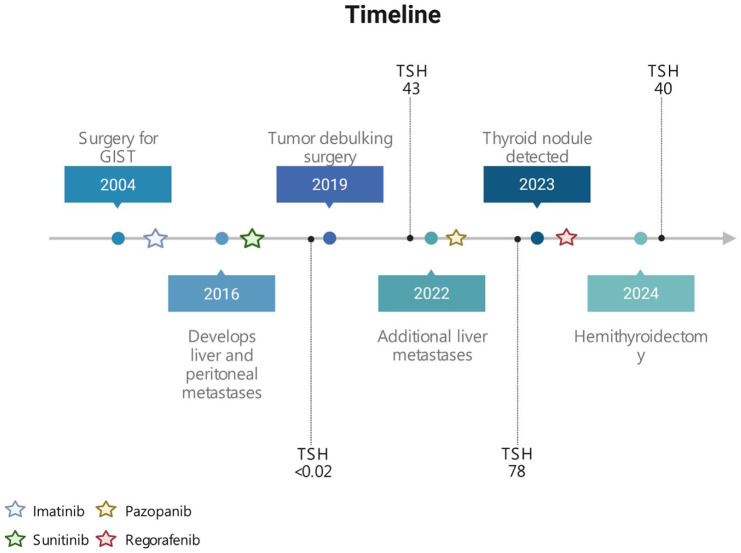
Timeline with important events during the clinical course of the patient. TSH levels are measured in serum (mU/L, normal reference 0.3–4.2 mU/L). TSH: Thyroid-stimulating hormone. Created using BioRender.com.

A 58-year-old man initially presented with a 50 mm jejunal GIST (c-KIT exon 11 variant), resected in Syria 2004, followed by 5 years of adjuvant imatinib. He remained recurrence-free until 2016, when liver and peritoneal metastases were diagnosed in Sweden. Cytology confirmed relapse, and imatinib (800 mg) was resumed, later switched to sunitinib in 2018 due to progression. Sunitinib achieved radiological stabilization, but recurrent ileus required debulking surgery in 2019, with clinical benefit and partial radiological response. Progression with liver metastases in July 2022 prompted pazopanib, preferred over regorafenib for toxicity profile, yielding temporary stabilization. Subsequent progression with lymph node involvement led to third-line regorafenib in September 2023. The clinical course is summarized in Figure 1.

## Diagnostic assessment

**Figure 2.**
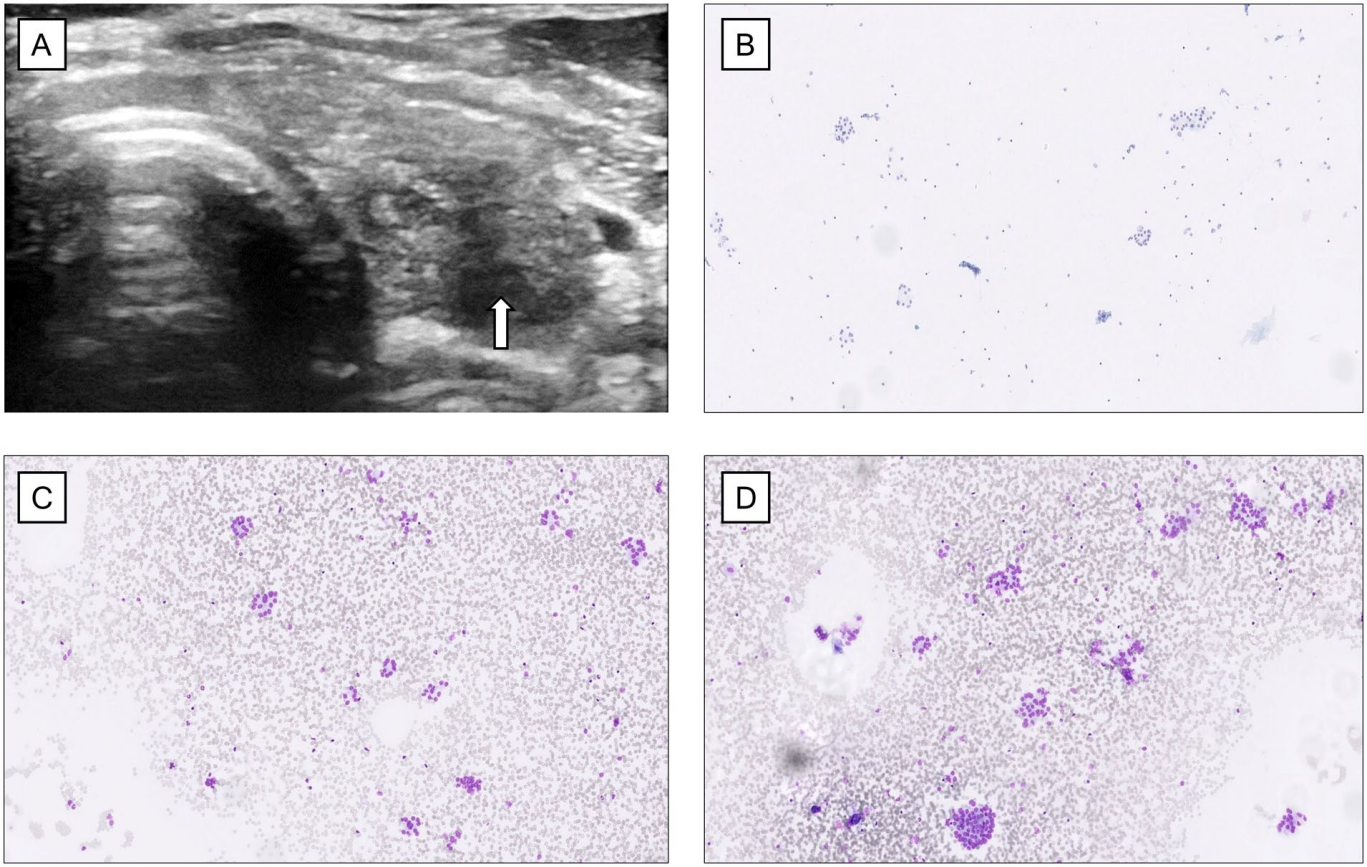
Ultrasound imaging of the left thyroid lobe revealed a well-demarcated area (white arrow) (a). Subsequent fine-needle aspiration cytology showed repetitive patterns of cells arranged in microfollicles, leading to a Bethesda IV category diagnosis (b–d).

**Figure 3.**
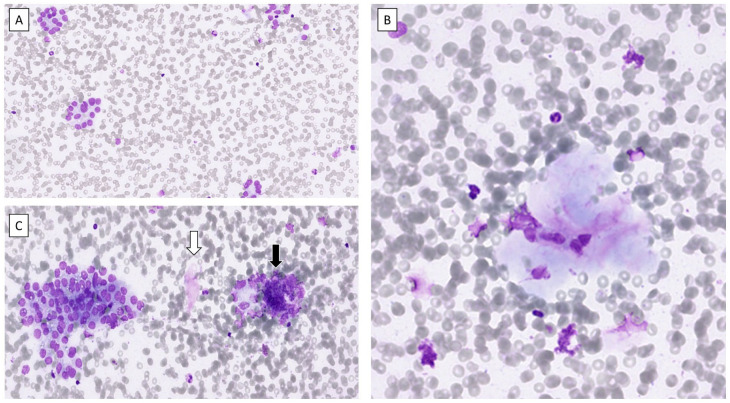
May-Grünwald Giemsa-stained preparations from the fine-needle aspiration biopsy of the enlarged thyroid. (a) Acinar structures composed of follicular cells were noted, and a Bethesda category IV diagnosis was favored. There was no nuclear atypia. (b) Hyaline material with entrapped epithelial cells was occasionally noted, which was also represented in histological preparations. (c) In other areas, similar findings of hyaline-like material (white arrow) were noted adjacent to clusters of crushed lymphoid cells (black arrow).

Due to a sensation of a lump in the throat, the patient underwent a physical examination in 2023. The thyroid was not painful upon palpation. The patient had no known family history of thyroid diseases. An ultrasound-guided fine-needle aspiration was performed in June of the same year, targeting a 17 mm suspected nodule (Thyroid Imaging Reporting and Data System category 5; TIRADS 5) located in the caudal part of the left thyroid lobe, resulting in a cytological diagnosis of suspicion for follicular neoplasm (Bethesda IV) (Figures 2 and 3). Imaging of the right thyroid lobe showed no suspicious findings. Furthermore, laboratory findings in 2018 indicated a suppressed TSH concentration of <0.02 μIU/mL (SI: <0.02 mIU/L) (reference range, 0.3–4.2 μIU/mL (SI: 0.3–4.2 mIU/L)), borderline free T4 (fT4) of 1.7 ng/dL (SI: 22 pmol/L) (reference range, 0.8–1.7 ng/dL (SI: 10–22 pmol/L)), and free T3 (fT3) of 3.6 pg/mL (SI: 5.6 pmol/L) (reference range, 2.1–4.2 pg/mL (SI: 3.3–6.0 pmol/L)). Between 2022 and 2024, the patient’s TSH concentrations were consistently elevated, ranging from 43 to 78 μIU/mL (SI: 43–78 mIU/L), while fT4 gradually decreased from 1.1 to 0.7 ng/dL (SI: 14.2–9.0 pmol/L) and fT3 from 2.7 to 2.3 pg/mL (SI: 4.2–3.5 pmol/L) (Figure 1). Anti-thyroid antibodies were not measured, likely due to a combination of clinical oversight and the prevailing suspicion that the hypothyroidism was TKI-induced rather than autoimmune in origin. The patient did not receive thyroid-specific treatment, as fT4 and fT3 concentrations remained within the normal range and he was clinically asymptomatic. Thyroid function was therefore managed with close biochemical monitoring. Although current guidelines often recommend levothyroxine therapy for TSH concentrations >10 mIU/L, treatment was deferred in this case given the absence of symptoms and the context of ongoing TKI therapy.

## Treatment

**Figure 4.**
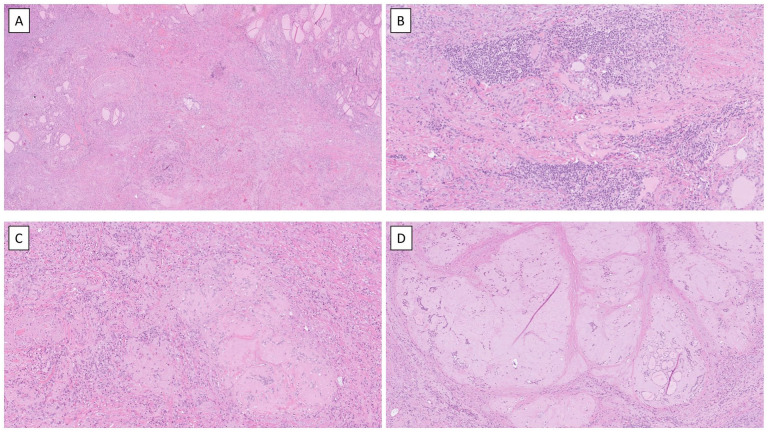
Histological hallmarks of TKI-induced thyroiditis. (a) Low-power overview of a hematoxylin and eosin-stained section from the thyroid lobe, illustrating the general lack of colloid-forming thyrocytes and replacement fibrosis. (b) Scattered lymphocytic infiltrates were noted; no secondary lymphoid structures were observed. Giant cells were absent. (c,d) Examples of the hyaline nodular structures that were commonly seen across the specimen, in low and high power, respectively. The nodules contained entrapped follicular cells positive for TTF1, PAX8 and thyroglobulin (not shown). TKI: Tyrosine kinase inhibitor.

In 2024, the patient underwent left hemithyroidectomy, with the gland noted as hard and adherent. Grossly, the lobe showed a fibro-elastic cut surface without a discernable tumor. Microscopically, normal thyroid tissue was largely replaced by fibrosis with nodular hyaline transformation, entrapped follicular cells, scattered lymphocytes, and degenerative areas with cholesterol crystal deposits (Figure 4). There was no follicular architecture with germinal centers, nor were giant cells identified. The overall histologic presentation therefore did not resemble chronic lymphocytic thyroiditis (Hashimoto thyroiditis) or giant cell thyroiditis; however, these features are not invariably present, and their absence does not definitively exclude either diagnosis. Given the dense fibrosis with hyalinization and entrapped follicles, Riedel’s thyroiditis and IgG4-related thyroid disease were considered in the differential diagnosis. In this case, the fibrosing inflammatory process was confined to the thyroid gland, with no extracapsular extension identified, and there was no evidence of phlebitis or a conspicuous plasma cell infiltrate on routine histology. Therefore, IgG/IgG4 immunohistochemistry was not pursued. The patient also had no clinical history or findings suggestive of IgG4-related disease in other organs. Focally, there were areas with remaining follicular arrangement exhibiting a compensatory hyperfunction; within these parts, up to 22 mitoses per 10 high-power fields and a Ki-67 index of nearly 30% were noted. However, nothing tumorous was observed. As expected, the intact parenchyma was positive for TTF1, PAX8-mono, and thyroglobulin, but negative for BRAF VE1, CK19 and HBME1. The inflammatory cells consisted of a mixed population, with CD20-positive B-lymphocytes in distinct clusters, while CD3-positive T cells were more dispersed, comprising both CD4-positive T helper cells and CD8-positive, cytotoxic T cells. The hyaline material did not stain for amyloid A component. Langerin identified only scattered Langerhans cells.

## Outcome and follow-up

Postoperatively, the patient’s well-being persisted, and although serum T4 concentrations remained within the normal range, TSH concentrations continued to be markedly elevated. This was likely due to pre-existing TKI-induced thyroid dysfunction, which may have impaired the compensatory function of the remaining thyroid tissue following hemithyroidectomy. Re-evaluation of the pre-operative fine-needle aspiration biopsy was performed in order to investigate if the rather unusual postoperative findings could be reproduced on the cytological level. Upon review, the cytology slides revealed a bloody smear with moderate cellularity and no colloid. The cellular composition was predominantly (>70%) follicular cells, accompanied by a smaller population of lymphoid cells. The follicular cells exhibited only partial oncocytic metaplasia. Anisonucleosis was present, but no nuclear atypia was observed. These epithelial cells were frequently arranged in follicular groups and acinar structures, with some of these groups showing infiltrating lymphocytes. The lymphoid population consisted mostly of small mature lymphocytes and occasional plasma cells. Small, crowded groups of lymphoid cells were appreciated, often showing crushing artifacts due to the fragile nature of these cells, a finding common in smears from thyroiditis (Figure 3). A few histiocytes, mesenchymal cells, and fragments of pink, hyaline-like stroma were also seen (Figure 3). The overall cytological picture was therefore not entirely typical for chronic lymphocytic thyroiditis, and the suspicion of follicular neoplasm could not be ruled out.

## Discussion

TKI-associated thyroid dysfunction is likely multifactorial and may involve direct follicular cell injury, impaired iodine uptake, altered thyroid hormone synthesis and metabolism, and increased peripheral hormone clearance.^5^ In some patients, a transient thyrotoxic phase may precede hypothyroidism, consistent with a destructive thyroiditis-like process. Clinically, current recommendations advocate baseline and periodic thyroid function testing during TKI therapy, as dysfunction may be dynamic. Overt hypothyroidism should be treated with levothyroxine, while selected asymptomatic patients with subclinical hypothyroidism may be managed with close biochemical surveillance without interrupting oncological treatment.

This case provides a unique opportunity to study the morphological attributes of a dysfunctional human thyroid gland from a patient who developed biochemically significant, largely asymptomatic hypothyroidism during treatment with the multikinase TKIs. The observed transient suppression of TSH with borderline elevated fT4 preceding the onset of hypothyroidism is in line with previous findings describing a biphasic course of TKI-induced thyroid dysfunction for many patients, in which an early thyrotoxic phase likely reflects follicular cell injury before progression to functional decline.^6^ The thyroid presented with a reduction in colloid-forming follicles, scattered areas of chronic inflammation, and widespread pale, infarction-like areas—a pattern not seen in other forms of thyroiditis. Typically, chronic lymphocytic thyroiditis presents with an abundant inflammatory infiltrate, often with secondary follicle formation, destruction of the thyroid parenchyma, and varying degrees of oncocytic metaplasia of the remaining thyroid follicular cells.^7^ In our case, none of these features were present on the histological level. Similarly, there were no multinuclear giant cells suggestive of de Quervain thyroiditis. The inflammatory response observed in the TKI-induced thyroiditis was mixed, with both B- and T-lymphocytes, the latter primarily composed of both CD4- and CD8-positive T cells. Plasma cells were not noted. This inflammatory profile is similar to chronic lymphocytic thyroiditis, although the histological pattern of this TKI-related thyroiditis is very different from what is normally observed microscopically in the former disease. It is also worth noting that this patient was diagnosed with a Bethesda IV category lesion (Figures 2 and 3), but no follicular neoplasm was evident in the histological preparations (Figure 4). However, some remaining thyroid follicular epithelial cells with regenerative properties were noted, which may explain the overall cytological pattern reported.

Given the increased attention to molecular testing in clinical pathology and the success of tailored oncological treatment schemes, the number of patients with TKI-induced hypothyroidism is expected to increase further. Therefore, awareness among clinicians as well as a fundamental understanding for practicing cytopathologists of the morphological appearance of this phenomenon is crucial. The occurrence of hyaline nodules with entrapped follicular cells may be a histological characteristic of TKI-induced thyroiditis (Figures 3 and 4), as these features are generally lacking in other forms of thyroiditis. Hyaline deposits are usually associated with hyalinizing trabecular tumors (HTTs) of the thyroid but may occasionally also be associated with papillary thyroid carcinoma.^8,9^ However, hyaline material in the setting of thyroiditis is not reported, apart from single reports of HTTs arising in a background of lymphocytic thyroiditis.^10^ Although we cannot establish the origin of these hyaline deposits with certainty, a plausible explanation might be the occurrence of widespread hypoxia and/or microinfarctions and subsequent tissue replacement, resulting from the intricate anti-angiogenic properties of TKIs.

At the time of decision-making, the 17 mm left thyroid nodule was classified as TIRADS 5 and Bethesda IV, raising concern for a follicular neoplasm. Despite the patient’s metastatic GIST and ongoing systemic therapy, the thyroid lesion was considered a potentially independent pathology. Although no radiological evidence of extrathyroidal extension or nodal disease was present, the combination of high-suspicion ultrasound features, indeterminate cytology, and local symptoms favored diagnostic hemithyroidectomy over surveillance, with acceptable surgical risk given the patient’s preserved general condition. Even so, the final histopathology showed no follicular thyroid tumor. This highlights the limitations of radiological and cytological systems, particularly in thyroiditis, where inflammation can mimic neoplasia. Re-evaluation of cytology confirmed frequent follicular cell groups consistent with Bethesda IV, underscoring the occasional, unavoidable discordance between cytology and final histology.

This report has several limitations. First, the timing of the histological alterations cannot be determined with certainty, though the changes are atypical for routine cases at our high-volume thyroid center. A review of 100 thyroidectomy specimens with Hashimoto thyroiditis revealed no similar hyaline structures (data not shown). Ideally, more thyroid samples from TKI-treated patients would be analyzed, but such cases are not yet available. It also remains unclear whether the histological findings were caused by a specific TKI or by their combination. Finally, anti-thyroid autoantibody testing was incomplete during both hyperthyroid and hypothyroid phases, although the histology does not match classic Hashimoto or Graves’ disease.

In summary, this case uniquely describes the cytological and histological features of possible TKI-induced hypothyroidism, marked by chronic lymphocytic infiltrates and hyaline elements with entrapped follicular cells. Further studies of thyroid resections in such patients may clarify the pathogenesis of this condition.

## Learning points

- TKI-induced hypothyroidism could possibly produce distinct histological changes not typically seen in other thyroiditis forms, including pale infarct-like areas and hyaline nodules with entrapped follicular cells.
- Lack of classic features such as germinal centers, oncocytic metaplasia, or giant cells could potentially distinguish this condition from Hashimoto or de Quervain thyroiditis.
- Cytology may mimic follicular neoplasia (Bethesda IV), but as no true neoplastic transformation was present during histological work-up, this emphasizes the need for cautious interpretation and correct clinical context.
- The presence of hyaline deposits in thyroiditis may reflect vascular injury or microinfarctions due to the anti-angiogenic effects of multikinase TKIs like sunitinib.
- Awareness of TKI-induced thyroid changes is essential for both clinicians and cytopathologists, especially as TKI use continues to expand in personalized oncology.
